# Cause-Specific Hospital Admissions on Hot Days in Sydney, Australia

**DOI:** 10.1371/journal.pone.0055459

**Published:** 2013-02-07

**Authors:** Pavla Vaneckova, Hilary Bambrick

**Affiliations:** School of Medicine, University of Western Sydney, Sydney, Australia; Johns Hopkins Bloomberg School of Public Health, United States of America

## Abstract

**Background:**

While morbidity outcomes for major disease categories during extreme heat have received increasing research attention, there has been very limited investigation at the level of specific disease subcategories.

**Methodology/Principal Findings:**

We analyzed daily hospital admissions for cardiovascular (CVD), respiratory (RD), genitourinary (GU) and mental diseases (MD), diabetes (DIA), dehydration (DEH) and ‘the effects of heat and light’ (HEAT) in Sydney between 1991 and 2009. We further investigated the sensitivity to heat of subcategories within the major disease groups. We defined hot days as those with temperatures in the 95^th^ and 99^th^ percentiles within the study period. We applied time-stratified case-crossover analysis to compare the hospital admissions on hot days with those on non-hot days matched by day of the week. We calculated the odds ratios (OR) of admissions between the two types of days, accounting for other environmental variables (relative humidity, ozone and particulate matter) and non-environmental trends (public and school holidays). On hot days, hospital admissions increased for all major categories except GU. This increase was not shared homogeneously across all diseases within a major category: within RD, only ‘other diseases of the respiratory system’ (includes pleurisy or empyema) increased significantly, while admissions for asthma decreased. Within MD, hospital admissions increased only for psychoses. Admissions due to some major categories increased one to three days after a hot day (e.g., DIA, RD and CVD) and on two and three consecutive days (e.g., HEAT and RD).

**Conclusions/Significance:**

High ambient temperatures were associated with increased hospital admissions for several disease categories, with some within-category variation. Future analyses should focus on subgroups within broad disease categories to pinpoint medical conditions most affected by ambient heat.

## Introduction

While morbidity outcomes for major disease categories during extreme heat have received increasing research attention, there has been very limited investigation at the level of specific disease subcategories [1]. Hot weather has been previously associated with significant increases in hospital admissions for cardiovascular, respiratory [2], [3], [4] and renal diseases [2], [5] heatstroke and heat exhaustion [2], [6], [7], dehydration, electrolyte imbalance, diabetes mellitus [7], [8], [9], nervous system disorders [2] and mental health [10].

Results of hospitalization studies were generally reported for all diseases combined, for broad disease categories such as cardiovascular or respiratory diseases [11], [12] or for some of their subsets [3], [4]. For example, an excess of total emergency hospitalizations was reported during the 2003 heatwave in England [13]. Also, Nitschke et al. reported an increase in total hospital admissions during heatwaves in Adelaide, Australia [14].

Excess hospitalizations due to broad specific categories have also been studied. An increase in hospitalizations due to all respiratory conditions was reported during hot days [9], [15]. Lin et al. reported an increase in hospital admissions due to total cardiovascular diseases among the elderly in New York City, USA [16]. When specific cardiovascular subsets were investigated, interesting information was revealed. For example, Koken et al. [17] found that while admissions due to acute myocardial infarction (AMI) and congestive heart failure increased with maximum temperature, during the same period the admissions due to coronary and pulmonary heart diseases did not increase [17]. While hospital admissions due to AMI, congestive heart disease and stroke increased for every 3°C among those aged 70+ in San Francisco and Sacramento [18], no relationship was reported between cerebrovascular diseases and hot weather in a study of twelve European cities [19]. This suggests that sensitivity to temperature may be disease-specific.

To date, the selection of specific morbidity outcomes for research studies has not been systematic; it was most likely driven by findings from mortality studies and from knowledge of physiological processes associated with extreme heat. Systematic studies that aim to determine which specific diseases within large disease groups are adversely affected by high ambient temperatures would greatly improve our understanding of how heat affects human health. Furthermore, such studies would reveal which population groups are most vulnerable and in need of preventive care during hot weather events.

For these reasons, in this project we analyzed hospital admissions in Sydney, Australia on unusually hot days for major disease groups and their subcategories.

## Data and Methods

### Ethics Statement

Under the Privacy Act 1988 legislation issued by the Office of Australian Information Commissioner, a written consent is generally sought for the collection of health information from a patient. The Privacy Act permits the handling of health information for health and medical research purposes in certain circumstances, where researchers are unable to seek individuals’ consent. The de-identified data originated from the Admitted Patient Data Collection (APDC) and were released by the NSW Health Department under a confidentiality agreement for the purposes of this study.

### Data Collection

**Figure 1:**
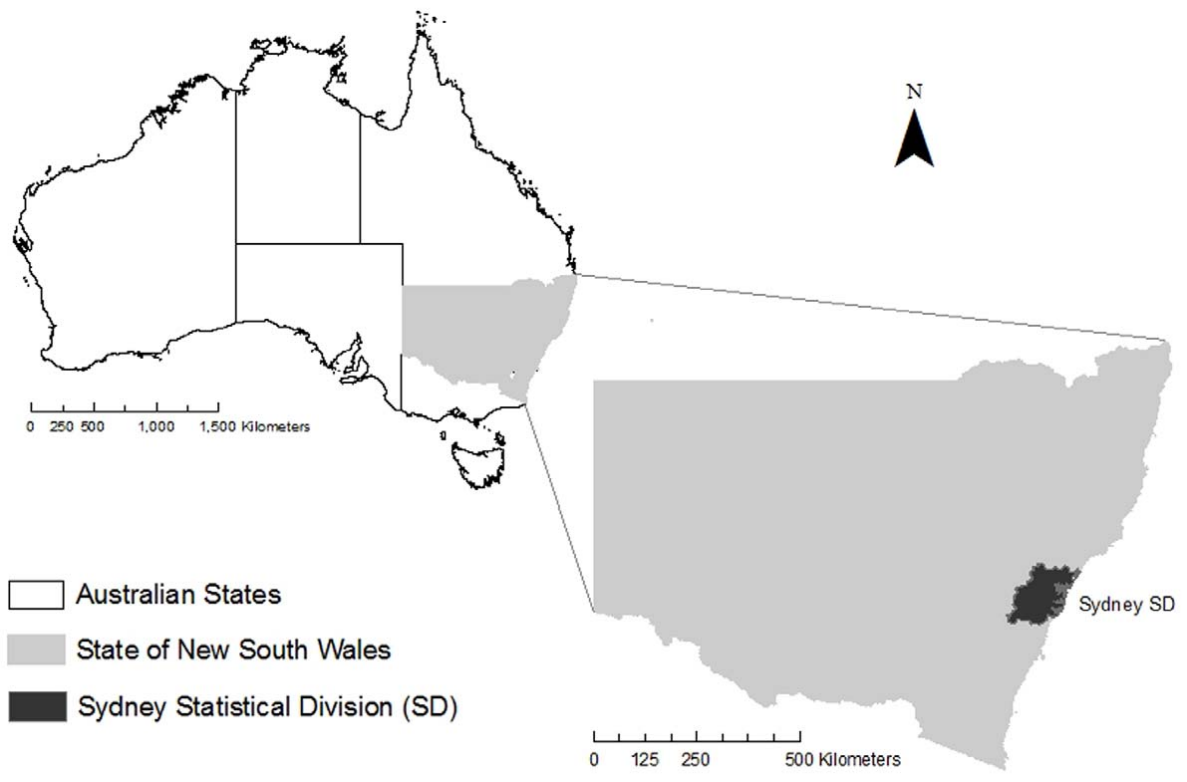
Location of the Sydney Statistical Division.

Sydney is the largest city in Australia and the capital of the state of New South Wales (NSW). It is situated on the southeast coast at 33.8°S latitude; its climate is mild temperate, with mild winters (average daytime maximum 16.3°C) and warm summers (average daytime maximum 25.9°C) [20]. The Sydney metropolitan area corresponds approximately to the Sydney Statistical Division (SD) administrative boundary, which occupies 12 145 km^2^, extending 90 km inland from the coast (Fig. 1). The Sydney SD had a population of 6.3 million in 2001 [21].

We obtained hospital admissions (emergency and planned) from all private and public hospitals located in the Sydney SD for the period July 1^st^ 1991- June 30^th^ 2009. The primary causes of admission were classified according to the International Classification of Diseases ICD9 code for the years 1991–1996, and following the ICD10 code for the period 1997–2009 (Appendix S1). As the use of an emergency flag is inconsistent within the dataset, with considerable differences between hospitals for example, we included all admissions in our analyses. We selected several broad disease classifications based on the literature as likely primary causes of hospital admission during hot days. These were cardiovascular (CVD), respiratory (RD), genitourinary (GU) and mental (MD) diseases, diabetes mellitus (DIA), volume depletion (hereafter dehydration, DEH), as well as the direct heat-related outcomes of the combined disease categories of ‘effects of heat and light’ and ‘exposure to natural heat’ (HEAT). We further divided the CVD, RD, GU and MD categories into more specific diagnoses and analyzed these separately. The selection of the broad groups of medical conditions was based on literature review; the choices of smaller subcategories were based on those that enabled a clear translation of the disease codes between the ICD9 and ICD10 classification through mapping tables developed and available at the National Casemix and Classification Centre at the University of Wollongong [22].

We obtained meteorological variables from the Australian Bureau of Meteorology (BOM). We used hourly measurements of temperature and relative humidity at four meteorological stations to calculate daily overall averages over the Sydney region. The following stations were selected because they are located within the Sydney SD, they measured continuously across the whole study period and each had less than 2% missing data: *Sydney Airport* (World Meteorological Organization (WMO) index number 94767, BOM station number 066037, 33. 9465°S, 150. 1731°E), *Bankstown* (WMO index number 94765, BOM station number 066137, 33. 9181°S, 150.9864°E), *Mount Boyce* (WMO index number 94743, BOM station number 063292, 33.6185°S, 150. 2741°E) and *Richmond RAAF* (WMO index number 95753, BOM station number 067105, 33.6004°S, 150.7761°E).

The NSW Environment Protection Authority provided hourly maximum concentrations of particulate matter of diameter less than 10 µm (PM_10_) and ozone (O_3_) for several monitoring stations across Sydney. We computed the daily average of all the stations within the Sydney SD that had less than 10% missing data, which comprised the three residential stations; namely, Richmond, Liverpool and Bringelly.

### Data Analysis

To identify ‘hot’ days, we first calculated daily values and obtained the probability distribution for the daily average temperature during the study period, identifying as ‘hot’ and ‘very hot’ those with temperatures within the highest 5 per cent and 1 per cent of the distribution.

We then applied the time-stratified case-crossover method [23]. The case-crossover design was proposed by Maclure [24] to study the effects of transient exposures on acute events, where the study population consists of only cases who also serve as their own controls. This method has been increasingly applied to epidemiological studies, especially for the investigation of ambient air pollution [25], [26]. It is as an alternative to time-series analysis, with almost identical results [27]. In our study, the time-stratified method divides the whole study period into equally sized, non-overlapping sections of 28 days (strata). When a case day falls within a stratum, it is then matched with control days within the strata by day of the week (for example, if a case day fell on a Monday, it was matched with the two or three Monday control days present within the 28-day stratum). The length of the strata is chosen so that it is short enough to remove the seasonal trend, but long enough to avoid correlation between the case and control days [28].

We then used conditional logistic regression to calculate the odds ratio (ORs) for hospital admissions on case days compared with controls days. The dependent variable was a hospital admission; the independent variables included the indicator of the extreme day (as a categorical variable), the daily relative humidity and the two air pollutants (O_3_ and PM_10_) as potential modifiers/confounders. Daily measurements of the two air pollutants were added to the model as linear independent variables, first separately and then jointly. We also included categorical variables for public and school holidays. The admission was then weighted by the frequency of all admissions on that day.

Since previous studies have reported a lagged and/or cumulative effect of extreme temperatures on health [15], [29], we also analysed the health outcomes 1–3 days after a hot day and on two and three consecutive days. Control days were chosen accordingly (admissions on control days for lag 1 to 3 were also lagged, and admissions on control days for 2 and 3 consecutive days were averaged for 2 and 3 consecutive days) to match by day of the week.

All p-values of the logistic regression test were adjusted for multiple testing using the False Discovery Rate (FDR) adjustment method [30].

## Results

Table 1 shows the daily descriptive statistics of the hospital admissions for the selected diseases. There were 12 disease categories for CVDs, nine for GUs, eight for RDs, four for MDs, one for DIA, one for DEH and one combined category for HEAT; a total of 36 categories. The largest number of hospital admissions was due to CVD (38%), followed by RD (26%), MD (23%), GU (10%), DIA (2%), DEH (0.4%), and HEAT (0.02%).

**Table 1:** Summary of hospital admissions by selected diseases between July 1^st^ 1991 and June 30^th^ 2009 in the Sydney Statistical Division.

	Total	Daily Mean	Min	Max	SD
**Respiratory disease, total**	**1,079,613**	**164.7**	**41**	**374**	**53.1**
Acute respiratory infections	164,794	25.1	2	83	11.6
Other diseases of the upper respiratory tract	259,506	39.5	0	126	27.3
Pneumonia and influenza	187,158	28.5	1	101	12.2
Chronic obstructive pulmonary disease and allied conditions	344,471	52.4	8	134	15.1
Asthma	181,819	27.7	2	113	12.2
Pneumoconioses and other lung diseases due to external agents	22,386	3.4	0	18	2.6
Other diseases of respiratory system	101,298	15.4	0	48	6.5
**Cardiovascular disease, total**	**1,561,231**	**225.4**	**56**	**371**	**76.9**
Acute rheumatic fever	325	0.04	0	2	0.2
Chronic rheumatic heart disease	4,553	0.7	0	7	1.0
Hypertensive disease	21,278	3.2	0	13	2.0
Ischaemic heart disease	543,433	82.7	10	157	27.8
Acute myocardial infarction	138,126	21.0	4	54	6.9
Diseases of pulmonary circulation	34,903	5.3	0	18	3.1
Other forms of heart disease	453,069	68.9	15	165	30.0
Cerebrovascular disease	166,463	25.3	2	51	6.5
Diseases of arteries, arterioles and capillaries	88,080	13.4	0	41	7.5
Diseases of veins and lymphatics	249,127	37.9	0	92	24.0
Other diseases of circulatory system	17,360	2.6	0	13	1.9
**Diseases of the genitourinary system, total**	**428,839**	**65.2**	**5**	**147**	**31.5**
Nephritis, nephrotic syndrome and nephrosis	121,245	18.4	0	49	8.4
Renal failure	20,914	3.2	0	12	2.1
Acute Renal Failure	10,362	1.6	0	9	1.4
Other urinary diseases	259,894	39.5	2	92	20.4
Genital organs male	31,710	4.8	0	22	4.0
Breast disorder	861	0.1	0	3	0.4
Inflammatory pelvic female	987	0.2	0	3	0.4
Other diseases of female genital tract	14,142	2.2	0	14	2.4
**Mental disorders, total**	**930,322**	**148.3**	**21**	**338**	**67.7**
Psychoses	420,586	64.0	6	165	26.3
Mental retardation	743	0.1	0	3	0.3
Neurosis	508,993	77.4	8	169	36.8
**Volume depletion (Dehydration)**	**16,692**	**2.5**	**0**	**16**	**1.9**
**Diabetes Mellitus**	**97,418**	**14.8**	**0**	**73**	**15.8**
**Effects of heat and light & Excessive heat (due to weather**	**997**	**0.2**	**0**	**16**	**0.6**
**conditions) combined**					

Within the CVDs, the most frequent admissions were due to ischemic heart disease (35%) and the least frequent were due to acute rheumatic fever (0.02%), which had the fewest presentations among all disease categories. The highest number of admissions among the RDs was for ‘chronic obstructive pulmonary disease and allied conditions’ (32%) and the lowest was for ‘pneumoconioses and other lung diseases due to external agents’ (2%). Among all GUs the ‘other urinary diseases’ (61%) was the category with the highest number of admissions, and ‘breast disorder’ had the fewest (0.2%). Among the three mental disorders, ‘neurosis’ (55%) and ‘psychoses’ (45%) accounted for most of the admissions, respectively, while mental retardation (0.08%) produced the fewest admissions.

Table 2 shows the daily statistics for the meteorological and air pollutant variables. From all the days in the study period (n = 6,575), 329 and 66 days fell into the highest 5 and 1% respectively, with average temperature thresholds of 24.4 and 26.4°C. On all case days, the average temperature was higher and the relative humidity was lower than on the control days. The levels of both air pollutants, O_3_ and PM_10_, were higher on case days.

**Table 2:** Average of temperature, humidity and air pollutants on days that were equal to or above the 95^th^ and 99^th^ percentile of daily average temperature distribution in the Sydney Statistical Division between July 1^st^, 1991 and June 30^th^, 2009.

	Average temperature (°C)		Relative humidity (%)		O_3_ (ppb)		PM_10_ (µg/m^3^)			
	Case	Control	Case	Control	Case	Control	Case	Control		
95^th^ percentile	25.3	20.4	58.6	66.9	2.6	1.5	27.5		19.3	
99^th^ percentile	27.5	20.4	50.5	61.7	2.9	1.4	33.1		20.0	

### Case Day Analysis

Table 3 shows the odds ratios (ORs) of hospital admissions during extremely hot days compared with control days. There is a statistically significant increase in the ORs of hospital admissions for RD, CVD, MD, and DEH on hot days (95^th^ percentile), and for DIA and HEAT on both hot and very hot days (95^th^ and 99^th^ percentiles).

**Table 3:** Odds ratios (adjusted for relative humidity, O_3_ and PM_10_) comparing hospital admissions due to several specific diseases between extremely hot days and control days in the Sydney Statistical Division between July 1^st^, 1991 and June 30^th^, 2009.

	95 percentile		99 percentile	
	OR	95% CI	OR	95% CI
**Respiratory disease, total**	**1.02**	**1.01–1.04**	1.02	0.99–1.04
Acute respiratory infections	*1.04	*1.01–1.08	1.02	0.95–1.10
Other diseases of the upper respiratory tract	1.03	1.00–1.05	1.04	0.99–1.09
Pneumonia and influenza	1.03	1.00–1.07	1.02	0.96–1.09
Chronic obstructive pulmonary disease and allied conditions	1.01	0.98–1.03	0.98	0.94–1.02
Asthma	0.97	0.93–1.00	**0.88**	**0.83–0.94**
Pneumoconioses and other lung diseases due to external agents	1.08	0.99–1.17	0.99	0.84–1.15
Other diseases of respiratory system	1.02	0.98–1.06	**1.11**	**1.02–1.20**
**Cardiovascular disease, total**	**1.01**	**1.00–1.02**	1.00	0.98–1.03
Acute rheumatic fever	1.09	0.56–2.11	2.10	0.57–7.67
Chronic rheumatic heart disease	0.87	0.70–1.07	0.95	0.63–1.43
Hypertensive disease	1.02	0.93–1.12	1.12	0.94–1.33
Ischaemic heart disease	1.00	0.98–1.02	1.00	0.97–1.04
Acute myocardial infarction	1.00	0.97–1.04	0.93	0.87–1.00
Diseases of pulmonary circulation	1.02	0.95–1.09	0.90	0.79–1.03
Other forms of heart disease	1.02	1.00–1.04	0.99	0.96–1.03
Cerebrovascular disease	0.99	0.96–1.02	0.95	0.90–1.01
Diseases of arteries, arterioles and capillaries	1.05	1.00–1.10	1.04	0.95–1.13
Diseases of veins and lymphatics	1.03	1.00–1.06	*1.07	*1.02–1.13
Other diseases of circulatory system	1.06	0.97–1.16	1.00	0.84–1.19
**Diseases of the genitourinary system, total**	1.00	0.98–1.02	1.03	0.99–1.07
Nephritis, nephrotic syndrome and nephrosis	1.02	0.98–1.05	1.01	0.94–1.08
Renal failure	1.05	0.96–1.14	0.98	0.84–1.15
Acute renal failure	1.06	0.94–1.19	*1.25	*1.02–1.54
Other diseases of urinary system	1.00	0.98–1.03	1.04	0.99–1.09
Diseases of male genital organs	0.97	0.89–1.04	1.03	0.89–1.19
Disorders of breast	1.00	0.67–1.49	0.43	0.19–0.97
Inflammatory disease of female pelvic organs	0.97	0.65–1.46	1.47	0.68–3.14
Other disorders of female genital tract	0.99	0.88–1.10	1.13	0.94–1.37
**Mental disorders, total**	**1.02**	**1.00–1.03**	1.01	0.98–1.03
Psychoses	**1.03**	**1.01–1.05**	1.03	1.00–1.07
Neurotic disorders, personality disorders, and other nonpsychotic mental disorders	1.01	0.99–1.02	1.00	0.97–1.04
Mental retardation	1.27	0.81–1.98	1.44	0.62–3.36
**Diabetes mellitus**	**1.06**	**1.02–1.10**	**1.12**	**1.04–1.20**
**Volume depletion (Dehydration)**	**1.18**	**1.09–1.29**	*1.19	*1.02–1.39
**Effects of heat and light & Excessive heat (due to weather conditions) combined**	**3.29**	**2.52–4.30**	**3.86**	**2.74–5.44**

Statistically significant values are in bold.

* OR became non-significant after multiple-testing adjustment.

Within all RDs, the admissions on very hot days (99^th^ percentile) for ‘other diseases of the respiratory system’ increased significantly, while the admissions for ‘asthma’ decreased significantly. While admissions due to all CVDs increased on hot days (95^th^ percentile), none of the CVD-specific diagnoses changed significantly. Among the admissions due to MDs, only admissions due to ‘psychoses’ increased significantly on hot days (95^th^ percentile). Against our expectations, the admissions due to GUs did not change significantly on hot and very hot days, neither as a major disease group nor in any of its specific disease subgroups.

### Lag Analysis

**Figure 2:**
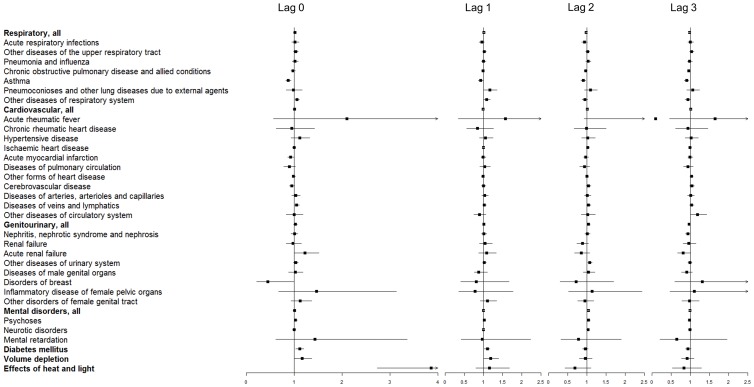
Odds ratios (adjusted for relative humidity, O_3_ and PM_10_) comparing hospital admissions due to several specific diseases between extremely hot days and control days in the Sydney Statistical Division between July 1^st^, 1991 and June 30^th^, 2009; on a hot day and 1, 2, and 3 days after the hot day at the *95^th^ percentile* of average temperature (results shown after the FDR adjustment). Note: the x-axis scale for admissions on hot days is different.

**Figure 3:**
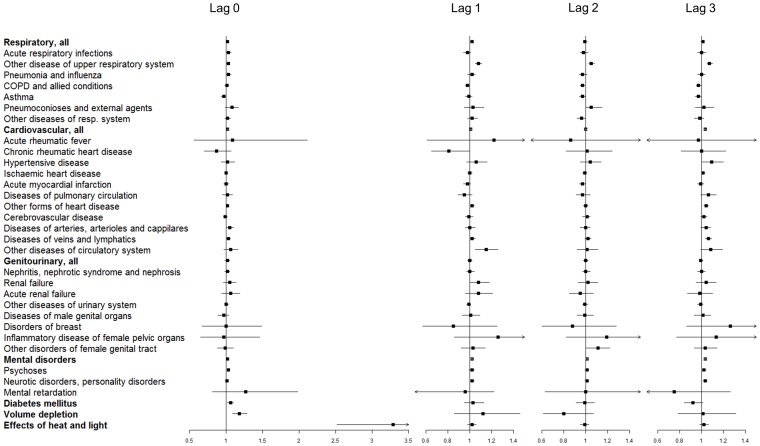
Odds ratios (adjusted for relative humidity, O_3_ and PM_10_) comparing hospital admissions due to several specific diseases between extremely hot days and control days in the Sydney Statistical Division between July 1^st^, 1991 and June 30^th^, 2009; on a hot day and 1, 2, and 3 days after the hot day at the *99^th^ percentile* of average temperature (results shown after the FDR adjustment). Note: the x-axis scale for admissions on hot days is different.

One day after a hot day, admissions for the broad category of RD remained significantly higher than on control days (95^th^ percentile; Fig. 2). Out of all the RDs, significantly higher admissions were detected for ‘other diseases of the upper respiratory tract’ one to three days after a hot day (95^th^ percentile; Fig. 2). Conversely, the admissions due to ‘chronic obstructive pulmonary disease and allied conditions’ decreased significantly two days after a hot day (95^th^ percentile), and ‘asthma’ remained significantly lower two and three days after a very hot day (99^th^ percentile; Fig. 3).

CVD, as a whole group, resulted in significantly higher admissions the third day after a hot day (95^th^ percentile; Fig. 2). When we analysed specific CVDs, admissions due to ‘other diseases of the circulatory system’ were significantly higher one day after, and admissions due to ‘other forms of heart disease’ and ‘diseases of veins and lymphatics’ three days after a hot day (Fig. 2). Hospital admissions due to all MDs were significantly higher one (95^th^ percentile), two (99^th^ percentile) and three (95^th^ percentile) days after a hot or very hot day. Among the specific MDs, the ‘psychoses’ and the ‘neurotic disorders, personality disorders, and other nonpsychotic mental disorders’ were significantly higher one and three days after a hot day (95^th^ percentile), respectively. Within the GUs, only admissions due to ‘other diseases of the urinary system’ increased significantly two days after a very hot day (Fig. 3). Admissions due to DIA were significantly higher one day after a very hot day (99^th^ percentile; Fig. 3), and admissions due to DEH and HEAT were not significantly different one to three days after a hot or very hot day (Fig. 2 and 3, respectively).

### Consecutive Days

Admissions on two and three consecutive hot days increased for all RDs (95^th^ percentile) and specifically for ‘pneumonia and influenza’ (95^th^ percentile). Conversely, admissions due to ‘asthma’ were significantly lower during two consecutive hot and very hot days. Among the MDs, three consecutive hot days (95^th^ percentile) were associated with increased admissions due to ‘neurotic disorders, personality disorders’ and ‘mental retardation’; and the admissions due to ‘psychoses’ increased significantly on two consecutive very hot days (99^th^ percentile). Admissions due to HEAT remained significantly higher on two and three consecutive days at the 95^th^ percentile. DEH was significantly higher on three consecutive hot days (95^th^ percentile).

### Multiple Testing

The FDR method was used to adjust for multiple testing and the effects on several specific disease subcategories became non-significant after the adjustment: on hot days, acute respiratory infections (95^th^ percentile, RD), diseases of veins and lymphatics (99^th^ percentile, CVD), acute renal failure (99^th^ percentile, GU) and DEH (99^th^ percentile). The effects on admissions for several other medical conditions became non-significant one to three days after hot days, such as DEH (99^th^ percentile), GU (99^th^ percentile), and some RD, CVD and GU disease subcategories (Fig. 2 and 3).

## Discussion

The aim of this study was to examine the impact of hot weather on specific causes of hospital admissions in Sydney, Australia. On a hot day, admissions increased in the broad categories of respiratory (RD), cardiovascular (CVD), mental (MD) diseases, and for diabetes (DIA), dehydration (DEH) and the ‘effects of heat and light’ (HEAT). Previous studies found that admissions for genitourinary (GU) (in particular, acute renal failure) tend to be higher on hot days [2], [5]; however, we did not find this association in our study. When examining whether people with specific disease subcategories were susceptible to extreme temperatures, we found that within all RDs only admissions for ‘other diseases of the respiratory system’ increased significantly, while those for ‘asthma’ decreased, and admissions due to the remaining RDs did not change. Within all MDs, only admissions due to ‘psychoses’ were higher. Admissions for CVDs and GUs did not change for any of the subcategories in relation to hot days.

Most morbidity studies to date have used large aggregated disease groups, such as all RDs or all CVDs [11], [12]; others focused only on one [4] or several specific health outcomes [18], [31]. The goal of our study was to analyze systematically the subcategories within broad disease classifications for non-fatal health outcomes. We hypothesized that when hospital admissions on hot days are aggregated into a large group (e.g., all CVDs) this may mask potential heat-morbidity relationships that occur for more specific outcomes.

Hospital admissions due to heat exhaustion or heatstroke, dehydration and electrolyte disorders can increase substantially during a heatwave [2], [7], [32]. During the 2006 heatwave in California hospital admissions increased significantly and the admissions increased 10-fold for heat-related illnesses (i.e., effects of heat and light, which include heat exhaustion and heatstroke) [8]. Semenza et al. [2] reported that approximately 62% of the excess hospital admissions during the 1995 Chicago heatwave were due to heatstroke, heat exhaustion or dehydration. In Australia, there was a 14-fold increase in direct heat-related admissions during the 2009 heatwave in Adelaide [33]. Khalaj et al. reported 590% increase in emergency hospital admissions due to heat-related injuries in five regions of New South Wales (including Sydney) [7]. In our study, hospital admissions due to heatstroke and heat exhaustion were approximately three times more likely when the average temperature was more than 24.3°C and the probability increased further if the temperature was more than 26.3°C (although the confidence intervals were also larger).

Dehydration is characterized by a loss of vital levels of liquids through sweating, urine (due to diuretics or uncontrolled diabetes), vomiting and diarrhea [34]. If not treated, dehydration can lead to a life-threatening emergency. Young children and elderly people are at higher risk of dehydration. Young children are vulnerable due to their lower weight and faster turnover of water and electrolytes [35]. The elderly are also more susceptible to dehydration due to age-related changes in total body water, thirst perception, and renal concentrating ability. Dehydration in older populations can also be related to infection, high-protein tube feedings, cerebral vascular accidents, and medication-related diminished thirst [34]. Dehydration was associated with increased hospital admissions in previous studies [2], [7], [9]. In our study the increase was significant; this risk could have been higher for some specific age groups.

Others have reported increases in hospital admissions due to renal diseases [8], [14]. Hansen et al. [5] found that hospital admissions for renal diseases and for acute renal failure were significantly higher during heatwaves compared with non-heatwave periods in Adelaide, Australia. In general, we did not find a relationship between ambient temperature and hospitalizations for renal disease, although admissions for acute renal failure were significantly higher before we adjusted for multiple-testing. Khalaj et al. [7] investigated the hospital admissions due to renal diseases on hot days in Sydney and some adjacent areas and did not find increased admissions. A recent study in Adelaide reported that the effects of temperature on renal admissions were not significant after adjustments for O_3_ and PM_10_ [36].

Hot weather could aggravate pre-existing conditions, such as respiratory or cardiovascular diseases. Significant increases in hospital admissions due to RDs have been previously reported [15], [16], [37], [38], with relatively fewer studies reporting increased numbers of admissions for total CVDs [2], [3], [8]. CVDs may be more acute than respiratory diseases, and result in death before help can be reached [38]. In Chicago, during the 1995 heatwave, CVDs increased only as an underlying rather than a primary cause of admission [2]. Some medications commonly prescribed for patients with heart disease, such as diuretics, could aggravate the effect of heat waves by provoking dehydration and electrolytic disorders [5]. CVDs may therefore contribute to the impact indirectly, rather than being the primary cause.

We did not find evidence of higher admissions due to any CVD subcategory on the day of the extreme heat event. This is in disagreement with some previous studies that reported increases in ischemic heart disease and in acute myocardial infarction [4], [39]. Some have recorded an increase in admissions due to CVDs for the elderly [40]. Our analyses were aggregated to all ages; it is therefore possible that admissions in the elderly age group may have increased during the hot weather events, without changes to the overall admissions due to CVD. Future analyses of CVD admissions focused on the elderly may help clarify this issue.

Within all RDs, only the subcategories of admissions for ‘other diseases of the respiratory system’ increased significantly, while those for ‘asthma’ decreased significantly; and admissions due to the remaining RDs did not change. The ‘other diseases of the respiratory system’ consist of empyema, pleurisy, pneumothorax, abscess of lungs and mediastinum. Empyema, pleurisy and abscess of lungs are characteristic for some types of infection that can develop in people with chronic underlying conditions. Infections can worsen during extremely hot weather [41]. Lower admissions due to asthma could be potentially due to people remaining in air conditioned environments during extremely hot days and therefore being exposed to lower levels of airborne allergens.

In our study, the ORs of admissions due to all mental diseases on a hot day were lower than the ORs of psychoses only, while ORs of admissions due to the other two mental disease subgroups (neurosis and mental retardation) were not significant. When admissions due to psychoses were removed from the total MD, the ORs of the remaining MDs aggregated became insignificant; therefore only those with psychotic disorders seemed more affected during the hot days in Sydney.

People with mental health problems have been found at higher risk during hot weather [10], [42], [43], [44], [45]. This could be due to their relatively poorer health, certain behavioral and social issues (e.g., substance misuse, socio-economic deprivation, living isolated or in institutional care), and the use of some psychotropic medications (e.g., antipsychotics, antidepressants and hypnotics) [6], [46], [47], [48]. Antipsychotics can interfere with physiological homestasis by altering the sweat threshold, impairing sweating and directly inducing hyperthermia [47]. A recent study in England found that antipsychotic medications were likely to play a more important role in the risk of heat injury than the psychosis condition per se [49]. The nosological subcategory of psychosis includes, among others, dementias and episodic mood disorders, which were also associated with hotter weather in Adelaide, Australia [10]. However, the same study also reported higher admissions due to neurotic, stress-related, somatoform disorders and disorders of psychological development on heatwave days, which we found non-significant. The psychoses in our study included schizophrenic disorders, which Hansen et al. [10] found non-significant. It is therefore possible that only some subcategories within psychoses are more affected by heat. If confirmed in future studies, this could further help focus the preventive care.

Previous morbidity studies have reported lagged impacts of extremely hot weather for some medical conditions [16], [29]. In our study, admissions for some medical conditions increased on the hot day and also one to three days later; while others did not increase on a hot day, but only three days after (MD: ‘neurotic disorders, personality disorders, and other nonpsychotic mental disorders’; CVD: ‘other forms of heart disease’ and ‘disease of veins and lymphatics’). Other conditions did not show a lagged effect (DEH and HEAT), and only increased on the hot day. Dehydration and heat-related illnesses seem to have a rather immediate medical urgency and may have affected younger, healthier populations that chose to engage in activities not suitable for hot weather (such as intense sports activities or outdoor hard work). Aging populations, more likely to suffer from several underlying pre-existing conditions and to have lower activity levels, may need a longer time to feel the impact of heat stress or could have been admitted to hospitals due to other medical conditions (such as RD, CVD and MD).

Several days of sustained high temperatures can be detrimental to health, due to lack of relief, especially when nocturnal temperatures remain high.There is evidence of higher morbidity levels with increased duration of an extreme event [15]. In our study, admissions due to some medical conditions such as DEH, HEAT and MDs increased when hot weather was sustained for two and three days. Consecutive second and third hot days also resulted in significant increases in admissions for RDs, specifically for pneumonia and influenza, while fewer admissions occurred for asthma.

Other studies have also noted a significant increase in primary admissions due to diabetes, suggesting that people with diabetes have an increased susceptibility to extreme heat [2], [8], [9], [50]. People with diabetes may have a greater risk due to an impaired ability to sweat and they may suffer from poorly controlled levels of blood sugars due to the fluid and electrolyte disturbances, which predisposes them to heat-related illness [2]. They are also more likely to suffer from other comorbidities (such as heart or renal disease). Others noted higher admissions when diabetes were analysed as an additional, rather than primary, cause [2].

A limitation in our study is that we used all hospital admissions, including both emergency and planned admissions. There is widely acknowledged inconsistency between years and between hospitals in the criteria used to determine emergency admissions in this dataset. Therefore, emergency admissions were not deemed superior to all admissions in our dataset. Our use of all admissions may have dampened the associations between heat and admissions as planned admissions were also included in the analyses. We would have expected some stronger associations if it had been possible to use a reliable set of unplanned admissions.

Another limitation is that our analysis was aggregated to a large metropolitan area, disregarding the spatial variation in temperature that exists within the region. A future study analyzing the spatial variation of temperature within the area would be beneficial. Also, the whole population was used regardless of age and gender. This was done to increase statistical power and to gain an overall impression of the non-fatal impacts of hot weather in Sydney. Age and gender are potential modifiers of the effect of heat on morbidity [1]; we intend to examine their contribution in a future study.

Another potential limitation is the use of average temperature as a heat indicator. Others have used various measures to identify heat vulnerability on populations, such as maximum, minimum or apparent temperatures [7], [11], [19], [36]. We believe that average temperature reflects the overall level of temperature exposure. In Sydney, rapid changes in temperature are possible due to a meteorological phenomenon called the ‘Southerly buster’, during which high temperatures are followed by a sudden, substantial drop. This change in weather offers relief for the Sydney population and would not be accounted for if maximum temperatures had been used. Previous studies have shown that average temperature is a good indicator of heat exposure [51], [52].

Projections of future climate point towards an increase in global temperatures and temperature extremes in Sydney and across Australia; the number of hot days and warm nights leading to reduced relief is also likely to increase [53]. Given that episodes of hot weather are likely to increase in the near future, it is vital to understand the potential vulnerability of local populations both for prevention and health service planning and response under such scenarios. This study enhances the existing knowledge of the effect of extremely hot temperatures on non-fatal outcomes.

Our results demonstrate that studies of temperature-morbidity relationships using broad disease classifications can underestimate the health impacts of hot weather events. Importantly for public health, analyses at the subcategory level would also help us to determine which patient groups are more vulnerable to extreme ambient temperature, so that resources for prevention (including education and surveillance) can be directed appropriately. Additional studies in other populations could improve the identification of the disease subgroups affected by heat and increase the evidence base so that suitable recommendations for both prevention and health service response can be made to local health departments, thus increasing the effectiveness of preventive and remedial care.

## Supporting Information

Appendix S1 **The selected specific causes of admissions.**
(DOC)
