# Supplementary material for: Cause-Specific Hospital Admissions on Hot Days in Sydney, Australia
Source: PLoS One. 2013 Feb 7;8(2):e55459. doi: 10.1371/journal.pone.0055459 (PMC3567089; doi:10.1371/journal.pone.0055459)
Supplement: Appendix S1 — The selected specific causes of admissions. (DOC) [file pone.0055459.s001.doc]

Appendix S1 The selected specific causes of admissions.

|  | **ICD9** |  | **ICD10** |
| --- | --- | --- | --- |
| **Cardiovascular diseases, all** | **390 - 459** |  | **I00 - I99** |
| Acute rheumatic fever | 390 - 392 |  | I00 – I02 |
| Chronic rheumatic heart disease | 393 - 398 |  | I05 –I07; I08.3-9; I09 |
| Hypertensive disease | 401 - 405 |  | I10 - I15 |
| Ischaemic heart disease | 410 – 414 |  | I20 - I22; I24 – I25.1- 9; |
| Acute myocardial infarction | 410 |  | I21- I22 |
| Diseases of pulmonary circulation | 415 - 417 |  | I26 – I28 |
| Other forms of heart disease | 420 - 429 |  | I23.8; I25.0; I30 – I52; I97.0,1,4; I98.0,1; |
| Cerebrovascular disease | 430 – 438 |  | I60 - I69; G45.0,4,8-9 |
| Diseases of arteries, arterioles and capillaries | 440 - 449 |  | I70 – I79, M30 - M31 |
| Diseases of veins and lymphatics | 451 - 457 |  | I80-I86; I89; I97.2; I98. 20,21; |
| Other diseases of circulatory system | 458 - 459 |  | I87; I95; I98.8,89; I99; R58 |
| **Respiratory diseases, all** | **460 - 519** |  | **J00 - J99** |
| Acute respiratory infections | 460 - 466 |  | J00 - J06, J20 - J21 |
| Other diseases of the upper respiratory tract | 470 - 478 |  | J30 – J39 |
| Pneumonia and influenza | 480 - 488 |  | J09 - J18 (excludes J18.2) |
| Chronic obstructive pulmonary disease and allied conditions | 490 - 496 |  | J40 – J47; J67 |
| Asthma | 493 |  | J45-46 |
| Pneumoconioses and other lung diseases due to external agents | 500 - 508 |  | J60 – J70 |
| Other diseases of respiratory system | 510 - 519 |  | J22; J80-J99(excludes J95.4,5,8,9); R09.1,8) |
| **Mental disorders** | **290 - 319** |  | **F00 - F99; G44.2** |
| Psychoses | 290-299 |  | F01.8; F02.8; F03; F05; F06; F09; F10.0, F10.4-10.7; F11.5- 11.9; F12.5- 12.9; F13.0, F13.3, F13.5; F15.0, F15.5; F16.5; F19.0, F19.3- 19.9; F20.0- 20.2, F20.5- 20.9; F21-22; F23.1- 23.9; F25.0- 25.9; F29; F30- 31; F32.0-32.3, F32.8-32.9; F33.0-33.4, F33.9; F38.8;  F39; F44.88; F44.9; F84.0-84.1,F84.3, F84.5, F84.8-84.9; |
| Neurotic disorders, personality disorders, and other nonpsychotic mental disorders | 300-316 |  | E34.9; F06.9; F07.0-07.2, F07.9; F10.0, F10.2; F11.1-11.2, F11.9; F12.1-12.2, F12.9; F13.1- 13.2, F13.9; F14.1-14.2, F14.9; F15.1-15.2, F15.9; F16.1-16.2, F16.9; F17.1,F17.9; F19.2, F19.9; F21; F32.9; F34.0**-** 34.8; F40.00, F40.01, F40.1-40.2, F40.9; F41.0- 41.1, F41.8- 41.9; F42.0, F42.8-42.9; F43; F44.0-44.1, F44.8- 44.9; F45.0, F45.2- 45.9; F48; F50.0, F50.2, F50.5, F50.8-50.9; F51-52; F54-55;F60; F63.0- 63.2, F63.8-63.9; F64.0-64.2; F65.0, F65.2-65.5, F65.8-65.9; F66.1, F66.9; F68.1; F69; F80.0-80.2, F80.8-80.9; F81.0-81.2, F81.8-81.9; F82-83; F88-89; F90; F91.1-91.9; F92.9; F93.0, F93.3-93.9; F94.0, F94.8; F95.0-95.2, F95.9; F98.0-98.5, F98.8; G44.2; L98.9; M62.99; |
| Mental retardation | 317-319 |  | F70.9, F71.9, F72.9, F73.9, F79.9 |
| **Diseases of the genitourinary system** | **580-629** |  | **N00-N99** |
| Nephritis, nephrotic syndrome and nephrosis | 580–599; 625.6; 788.31-5,7,9 |  | N01, N03-N05, N07-N09; N14, N15.0,2-9; N16-N19; N25-N27; N29.8 |
| Renal failure | 584 - 586 |  | N17 - 19 |
| Acute renal failure | 584 |  | N17 |
| Other diseases of urinary system | 590-599 |  | N02.9; N10-12; N13.0 -13.5; N13.7; N13.9; N15.1; N15.9; N16; N20.0-20.1; N20.9; N21; N28; N30; N31.2- 31.9; N32; N33.8; N34-36; N37.8; N39.0, N39.2, N39.88,N39.9; N99.1; R31 |
| Diseases of male genital organs | 600-608 |  | N40- 41; N42.0 - 42.2, N42.8 -42.9; N43- 47; N48.0 - 48.4; N48.6 - 48.9; N49.0, N49.9; N50; N51.0- 51.1, N51.8; |
| Disorders of breast | 610-612 |  | N60–62; N64 |
| Inflammatory disease of female pelvic organs | 614-616 |  | N70- 73; N75.0 - 75.1; N76.0 - 76.2, N76.4, N76.6 - 76.8; N77 |
| Other disorders of female genital tract | 617-629 |  | E23.0; N28.8; N32.2; N39.3; N80; N81.0- 81.5, N81.8- 81.9; N82; N83.0-83.5, N83.7-83.9; N84.0 - 84.3; N85.0, N85.2 - 85.9; N86; N87.9; N88; N89.0 - 89.6, N89.8 -89.9; N90.0 -90.6, N90.8 -90.9; N91-93; N94.0 - 94.3, N94.6-94.9; N95; N97.0 -97.3, N97.8 -97.9; N99.3 |
| **Diabetes mellitus** | **250** |  | **E10 - E11(or E14)** |
| **Volume depletion** | **276** |  | **E86 - E87** |
| **Effects of heat and light** | **992** |  | **T67** |
| **Excessive heat (due to weather conditions)** | **E900.0** |  | **X30** |
